# Controversial topics in metastatic HR+/HER2- breast cancer: Guiding treatment by a modified Delphi approach

**DOI:** 10.3389/fonc.2022.950861

**Published:** 2022-09-09

**Authors:** Alessandra Fabi, Giuseppe Buono, Emilio Bria, Giampaolo Bianchini, Giuseppe Curigliano, Michelino De Laurentiis, Sabino De Placido, Lucia Del Mastro, Valentina Guarneri, Daniele Generali, Lorenzo Livi, Vito Lorusso, Filippo Montemurro, Fabio Puglisi, Paolo Vigneri, Alberto Zambelli, Grazia Arpino

**Affiliations:** ^1^ Precision Medicine in Breast Cancer Unit, Scientific Directorate, Department of Woman and Child Health and Public Health, Division of Gynecologic Oncology, Fondazione Policlinico Universitario Agostino Gemelli IRCCS, Rome, Italy; ^2^ Department of Breast and Thoracic Oncology, National Cancer Institute, IRCCS Fondazione G Pascale, Naples, Italy; ^3^ Comprehensive Cancer Center, Fondazione Policlinico Universitario Agostino Gemelli IRCCS, Università Cattolica del Sacro Cuore, Rome, Italy; ^4^ Department of Medical Oncology, IRCCS Ospedale San Raffaele, Milan, Italy; ^5^ Department of Oncology and Hemato-Oncology, University of Milan, Milan, Italy; ^6^ Division of Early Drug Development, European Institute of Oncology, IRCCS, Milan, Italy; ^7^ Department of Clinical Medicine and Surgery, Oncology Division, University of Naples “Federico II”, Naples, Italy; ^8^ Department of Internal Medicine and Medical Specialties (DiMI), School of Medicine, University of Genova, Genova, Italy; ^9^ Department of Medical Oncology, Clnical Unit of Medical Oncology, IRCCS Hospital Policlinico San Martino, Genova, Italy; ^10^ Department of Surgery, Oncology and Gastroenterology, University of Padova, Istituto Oncologico Veneto (IOV) IRCCS, Padova, Italy; ^11^ Department of Medicine, Surgery and Health Sciences, University of Trieste, Trieste, Italy; ^12^ Department of Experimental and Clinical Biomedical Sciences “M. Serio”, University of Florence, Florence, Italy; ^13^ Radiation Oncology Unit, Oncology Department, Azienda Ospedaliero-Universitaria Careggi, Florence, Italy; ^14^ Unitá Operativa Complessa (U.O.C) Medical Oncology, IRCCS Istituto Tumori “Giovanni Paolo II”, Bari, Italy; ^15^ Breast Surgery Strategic Program, Candiolo Cancer Institute, Fondazione del Piemonte per l'Oncologia (Piedmont Foundation for Oncology) - IRCCS, Torino, Italy; ^16^ Department of Medical Oncology, Unit of Medical Oncology and Cancer Prevention, Centro di Riferimento Oncologico di Aviano (CRO) IRCCS, Aviano, Italy; ^17^ Department of Medicine (DAME), University of Udine, Udine, Italy; ^18^ Department of Clinical and Experimental Medicine, University of Catania, Catania, Italy; ^19^ Center of Experimental Oncology and Hematology, Azienda Ospedaliera Universitaria, Policlinico “G. Rodolico – San Marco”, Catania, Italy; ^20^ Department of Biomedical Sciences, Humanitas University, Milan, Italy; ^21^ IRCCS Humanitas Research Hospital, Milan, Italy

**Keywords:** metastatic HR+/HER2-breast cancer, Delphi survey, CDK4/6i, oncology, consensus

## Abstract

The treatment of HR+/HER2- metastatic breast cancer with cyclin-dependent kinases 4 and 6 inhibitors combined with endocrine therapy has recently emerged as the most relevant therapeutic strategy. However, in routine clinical practice, the best therapeutic approach in patients with comorbidities at early relapsing or *ab initio* metastatic disease, *PI3KCA* mutation, is still debated among oncologists. Given these areas of uncertainty, we conducted a Delphi survey to describe and confront the level of agreement or disagreement between clinicians working in referral vs local spoke oncological hospitals and summarize a consensus on these debated topics. In total, 56 items were drafted using the Nominal Group Technique and used for the Delphi Survey. A total of 46 clinicians participated in the survey. Overall, the consensus threshold among all participants was reached in 46/56 items (82%), and Delphi Survey results showed a high level of consensus. For the 10 items (18%) that did not reach the consensus threshold, possible explanations considering differences in clinical practice and recent findings from literature are provided in the Discussion. Outcomes from the present survey may help guide treatment in multiple comorbidities, early recurring and *ab initio* metastatic disease, and *PI3KCA* mutation, where evidence from randomized trials and level 1 evidence is currently missing.

## 1 Introduction

Hormone receptor-positive (HR+)/human epidermal growth factor receptor 2-negative (HER2-) metastatic breast cancer (MBC) accounts for approximately 65% of MBC cases (1–3). Treatment with cyclin-dependent kinases 4 and 6 inhibitors (CDK4/6i) – namely palbociclib, ribociclib, and abemaciclib – combined with endocrine therapy (ET) has recently emerged as the most relevant treatment in HR+/HER2- MBC (4–6) and is the recommended first-line strategy in this setting. Chemotherapy remains indicated for visceral crisis or rapidly progressive life-threatening disease (7–11).

The clinical efficacy of CDK4/6i has been widely proved in several clinical areas (12–20). However, in routine clinical practice, several topics, such as the role of comorbidities, the best approach to early relapsing or *ab initio* metastatic disease, and the part and timing of novel therapy, such as *PI3KCA* inhibitors, are still debated among oncologists.

The present study aims to highlight the major areas of uncertainty in this field, describe and confront the level of agreement or disagreement between clinicians working in a referral or local spoke oncological hospitals distributed all over the country, and summarize a consensus where possible.

## 2 Methods

**Figure 1:**
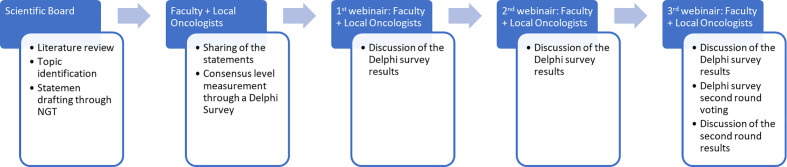
Project flowchart. NGT, nominal group technique.

The work structure is demonstrated in **Figure 1**. A scientific board (SB) reviewed the available literature and identified three topics of interest. The SB generated some statements within the issues through the Nominal Group Technique (NGT), then used an adapted Delphi survey. The Delphi survey was the answered by two groups of oncologists: the “local oncologists” and the “faculty” (i.e., an extended SB). Three subsequent meetings between the local oncologists and the faculty were used to discuss the submitted survey statements and results and participate in a second round of voting to address statements that did not reach consensus.

### 2.1 The Delphi panel: SB, faculty, and local oncologists

Overall, 42 clinicians formed the Delphi Panel.

The SB comprised four nationally and internationally recognized oncologists with extensive expertise in breast cancer who engaged in oncological research on breast cancer. One of the members served as a methodologist and facilitator to avail the SB in providing meeting facilitation, material preparation, and scientific accuracy. All SB members come from relevant Italian oncological hub centers for breast cancer.

Twenty-six Italian oncologists, who were experts in breast and other cancers, formed the “local oncologists” group. Unlike the SB, they come from either local oncological spoke centers or hub centers. Spokes are first-line referral centers, while hubs are second-line referral centers with more knowledge and expertise to which complex clinical cases are referred.

The faculty is an extended SB, and its members share the same inclusion criteria. Their presence served to counterbalance the number between the SB and local oncologists and to bring a wider perspective from experts on the topics discussed. On top of the four clinicians of the SB, the faculty included an additional 12, for a total of 16 clinicians.

### 2.2 Key topics choice and statement generation

Focusing on unclear criteria of therapeutic options for patients with HR+/HER2- MBC, the SB identified three topics of interest through literature review and discussion and personal clinical experience (1): first-line therapeutic choices in patients with HR+/HER2- MBC and multiple comorbidities; (2) first-line therapeutic choices in early recurring HR+/HER2- MBC patients; and (3) first-line therapeutic choices in *ab initio* HR+/HER2- MBC patients. These topics were chosen as they were considered to be debated among oncologists in routine clinical practice.

For every topic, some statements were defined by the SB using the NGT. The NGT is a direct and structured expert opinion-based technique aimed at managing organized meetings to make decisions on a specific topic with no strong evidence (21). By addressing the three key topics, SB members had the opportunity to independently develop their own thoughts and opinions. Their ideas were presented during an online meeting in January 2021, chaired by a professional facilitator. The views were collected and shared with the participants, who ranked them through an online survey in terms of priority and relevance using a 1–5 Likert scale. Similar opinions were then merged. Eventually, the complete list of items was drafted.

### 2.3 Modified Delphi process

After statements were finalized, a survey with all the statements was submitted and completed by the faculty and local oncologists through the Delphi Method. The Delphi Method is a well-established method of consensus used to evaluate the level of agreement (consensus quantification) and to resolve differences of opinion (consensus development) with a Likert scale (1–5; 1 = total disagreement; 2 = slight disagreement; 3 = partial agreement; 4 = agreement; 5 = total agreement). It takes place anonymously and interactively, often using online surveys, through several rounds or phases of evaluation and expression of opinions by a group of appropriately selected experts (22). The consensus was defined as reaching a level of agreement (defined as partially agree + agree + totally agree) or disagreement (defined as totally disagree + slightly disagree) ≥66.6% (i.e., two-thirds) between all participants. Instead of continuously voting until a final consensus was reached, as contemplated by the original methodology, the process was adapted by carrying out only two rounds of voting. The second round addressed only statements without consensus. To present the results during webinars and in this paper, the Likert scale was replaced with a binary scale (agree/disagree). The levels of agreement/disagreement between the faculty and local oncologists are shown separately to compare them (**Tables 1**–**3**).

**Table 1:** Level of agreement and disagreement and consensus status on statements regarding comorbidities.

	Level of agreement or disagreement		Consensus	
	Faculty	Local oncologists	Faculty	Local oncologists
*1. In patients with multiple comorbidities, the treatment with CDK4/6i in association with endocrine therapy (ET), in the absence of precise contraindications and visceral crisis, is to be considered the first-line standard treatment in the following clinical situations:*				
1.1. Exclusively bone disease	80%	100%	Yes, agreement	
1.2. Visceral disease with ≥3 metastatic sites	93%	100%	Yes, agreement	
1.3. Visceral disease, regardless of disease burden	100%	96%	Yes, agreement	
*2. In relation to the previous question, in clinical situations in which it was decided to use a CDK4/6i, the choice could fall on:*				
2.1. Abemaciclib	93%	100%	Yes, agreement	
2.2. Palbociclib	80%	92%	Yes, agreement	
2.3. Ribociclib	87%	100%	Yes, agreement	
*3. In patients with multiple comorbidities, should a reduction in the used dose of the CDK4/6i be considered from the start?*				
	88%	88%	Yes, disagreement	
*4. In the presence of multiple comorbidities in a very old patient (> 75 years old), should a reduction in the dose of the CDK4/6i used to be considered from the start?*				
	80%	88%	Yes, disagreement	
*5. In the presence of a very old patient (>75 years old), is there a preference for a CDK4/6i over the others?*				
	57%	60%	No	No
6. *In patients with multiple comorbidities and/or very elderly, is the choice of CDK4/6i to be made considering the different toxicity profiles and the different pharmacological interactions of the three drugs?*				
	100%	100%	Yes, agreement	
7. *In patients with multiple comorbidities, the evaluation of PIK3CA (on tissue or liquid biopsy) should be carried out:*				
7.1. At the first diagnosis of metastatic disease, so as to define the therapeutic path of first and second line	64%	92%	No	Yes, agreement
7.2. Before starting second-line therapy, if not previously performed	93%	96%	Yes, agreement	
7.3. Before starting the second-line therapy, even if previously carried out and negative	64%	64%	No	
*8. Can the research of the PIK3CA mutation using liquid biopsy be considered a valid subrogate with respect to the tissue in patients where this procedure is not feasible or has been rejected?*				
	93%	100%	Yes, agreement	
9. *In patients with multiple comorbidities, in the absence of* PIK3CA *mutation, is the first-line treatment with ribociclib in association with fulvestrant to be preferred over aromatase inhibitor (AI) + CDK4/6i?*				
	60%	64%	No	
10. *In patients with multiple comorbidities and with* PIK3CA *mutation, the treatment with alpelisib + fulvestrant, in the absence of specific contraindications, can be considered a standard treatment after progression by AI ± CDK4/6i?*				
	100%	96%	Yes, agreement	

Percentage in green indicates the level of agreement, and in red the level of disagreement. Consensus on agreement was reached if levels of agreement were >66%. Consensus on disagreement was reached if levels of disagreement were >66%. Items in yellow are those where consensus was not met by either the faculty or local oncologists.

**Table 2:** Level of agreement and disagreement and consensus status on statements regarding early relapse.

	Level of agreement or disagreement		Consensus	
	Faculty	Local oncologists	Faculty	Local oncologists
*1. In young, premenopausal patients at the beginning of adjuvant ET, with early recurrence (within 2 years from the beginning of ET), the treatment with luteinizing hormone-releasing hormone antagonist (aLHRH) + fulvestrant + CDK4/6i can be considered as first-line standard treatment, in the absence of visceral crisis, in the following clinical situations:*				
1.1. Exclusively bone disease	87%	92%	Yes, agreement	
1.2. Visceral disease with ≥3 metastatic sites	100%	96%	Yes, agreement	
1.3. Visceral disease, regardless of disease burden	100%	96%	Yes, agreement	
*2. In relation to the previous question, in clinical situations in which it was decided to use aLHRH + fulvestrant + CDK4/6i, the choice could fall on:*				
2.1. Abemaciclib	92%	80%	Yes, agreement	
2.2. Palbociclib	64%	52%	No	
2.3. Ribociclib	100%	88%	Yes, agreement	
*3. In young, premenopausal patients with early recurrence (within 2 years after the beginning of ET), and at the beginning of adjuvant ET, is a first-line chemotherapy treatment preferable over fulvestrant + CDK4/6i?*				
	67%	88%	Yes, disagreement	
*4. In young, premenopausal patients with early recurrence (within 2 years after the beginning of ET), at the beginning of adjuvant ET, and treated with tamoxifen monotherapy, may it be considered a first-line treatment with aLHRH + AI + CDK4/6i?*				
	80%	96%	Yes, agreement	
*5. In young, premenopausal patients with early recurrence (within two years after the beginning of ET), at the beginning of adjuvant ET, treated with tamoxifen monotherapy, and presenting* PIK3CA *mutation, may it be considered a first-line treatment with aLHRH + AI + CDK4/6i to allow a second-line treatment with fulvestrant + alpelisib?*				
	87%	100%	Yes, agreement	
6. *In young, premenopausal patients with early recurrence (within 2 years after the beginning of ET), at the beginning of adjuvant ET and candidate for CDK4/6i + ET, can the choice of ribociclib be considered preferential over palbociclib and abemaciclib?*				
	87%	100%	Yes, agreement	
7. *In postmenopausal patients with early recurrence (within two years after the beginning of ET), and not in visceral crisis, treatment with fulvestrant + CDK4/6i can be considered standard first-line treatment in the following clinical situations:*				
7.1. Only bone recurrence	93%	96%	Yes, agreement	
7.2. Visceral relapse with ≥3 metastatic sites	93%	96%	Yes, agreement	
7.3. Visceral relapse regardless of disease burden	100%	96%	Yes, agreement	
*8. In relation to the previous question, in clinical situations in which it was decided to use fulvestrant + CDK4/6i, the choice could fall on:*				
8.1. Abemaciclib	100%	92%	Yes, agreement	
8.2. Palbociclib	71%	64%	Yes, agreement	No
8.3. Ribociclib	100%	92%	Yes, agreement	
9. *In postmenopausal patients with early recurrence (within 2 years after the beginning of ET), and not in visceral crisis, is a first-line chemotherapy treatment preferable over fulvestrant + CDK4/6i?*				
	87%	92%	Yes, disagreement	
10. *In elderly patients (>75 years old) with early recurrence (within 2 years after the beginning of ET), and not in visceral crisis, a first-line monotherapy treatment with fulvestrant is preferable over its combination to CDK4/6i in the following clinical situations:*				
10.1. Only bone recurrence	67%	68%	Yes, disagreement	
10.2. Visceral relapse with ≥3 metastatic sites	87%	88%	Yes, disagreement	
10.3. Visceral relapse regardless of disease burden	87%	96%	Yes, disagreement	
*11. In elderly patients (>75 years old) with early recurrence (within 2 years after the beginning of ET), and not in visceral crisis, is a first-line chemotherapy treatment preferable over the fulvestrant + to CDK4/6i combination?*				
	93%	96%	Yes, disagreement	

Percentage in green indicates the level of agreement, and in red the level of disagreement. Consensus on agreement was reached if levels of agreement were >66%. Consensus on disagreement was reached if levels of disagreement were >66%. Items in yellow are those where consensus was not met by either the faculty or local oncologists.

**Table 3:** Level of agreement and disagreement and consensus status on statements regarding *ab initio* metastatic tumor.

	Level of agreement or disagreement		Consensus	
	Faculty	Local oncologists	Faculty	Local oncologists
*1. In* ab initio *metastatic patients, the first-line treatment with ET ± CDK4/6i is to be preferred over chemotherapy in case of:*				
1.1. Exclusively bone disease	93%	100%	Yes, agreement	
1.2. Oligometastatic disease	100%	100%	Yes, agreement	
1.3. Visceral disease, regardless of disease burden	100%	100%	Yes, agreement	
1.4. Visceral disease, with high disease burden	100%	92%	Yes, agreement	
*2. In* ab initio *metastatic patients, the first-line treatment with CDK4/6i + ET is to be preferred over ET only in case of:*				
2.1. Exclusively bone disease	93%	96%	Yes, agreement	
2.2. Oligometastatic disease	100%	100%	Yes, agreement	
2.3. Visceral disease, regardless of disease burden	100%	96%	Yes, agreement	
2.4. Visceral disease, with high disease burden	100%	96%	Yes, agreement	
*3. In 75+ years old*, ab initio *metastatic patients, the first-line treatment with CDK4/6i + ET is to be preferred over ET only in case of:*				
3.1. Exclusively bone disease	93%	88%	Yes, agreement	
3.2. Oligometastatic disease	100%	84%	Yes, agreement	
3.3. Visceral disease, regardless of disease burden	100%	100%	Yes, agreement	
3.4. Visceral disease, with high disease burden	100%	100%	Yes, agreement	
*4. In* ab initio *metastatic patients without* PIK3CA *mutations, the first-line treatment with fulvestrant + ribociclib combination is to be preferred over combination of AI + any CDK4/6i in case of:*				
4.1. Exclusively bone disease	64%	64%	No	No
4.2. Oligometastatic disease	50%	56%	No	No
4.3. Visceral disease, regardless of disease burden	64%	64%	No	No
4.4. Visceral disease, with high disease burden	64%	72%	No	Yes, agreement
*5. In case of visceral crisis and* ab initio *metastatic disease with high hormonal receptor expression, can an ET + CDK4/6i be proposed over a "rescue" chemotherapy?*				
	87%	64%	Yes, agreement	No
6. *In* ab initio *metastatic patients, should the search for the* PIK3CA *mutation (on tissue or liquid biopsy) be carried out before the start of the first-line therapy?*				
	93%	80%	Yes, agreement	
7. *In* ab initio *metastatic patients with* PIK3CA *mutation, should the therapeutic strategy include a first-line treatment with AI + CDK4/6i and a second-line one with fulvestrant + alpelisib, in the absence of specific contraindications?*				
	100%	100%	Yes, agreement	


Percentage in green indicates the level of agreement, and in red the level of disagreement. Consensus on agreement was reached if levels of agreement were >66%. Consensus on disagreement was reached if levels of disagreement were >66%. Items in yellow are those where consensus was not met by either the faculty or local oncologists.

### 2.4 Statistical analysis

All data were analyzed with descriptive statistics.

## 3 Results

### 3.1 Statement drafting and Delphi survey

During the NGT, the SB addressed the three key topics and drafted 28 statements: 10 for the first topic regarding first-line therapeutic choices in case of comorbidities, 11 for the second topic regarding first-line therapeutic choices in case of early relapse, and seven for the third one, which regarded first-line therapeutic choices in case of *ab initio* metastatic tumor. As some statements addressed the same problem in different clinical scenarios, three to four sub-options per statement were also included, resulting in 56 items to be voted. The generated statements for every key topic are shown in **Tables 1**–**3**.

All 42 clinicians participated in the Delphi survey. Consensus on agreement from both the local oncologists and the faculty was reached in 38 out of 56 (68%) items; consensus on disagreement from both the local oncologists and the faculty was achieved in eight (14%) items. In total, 46/56 options (82%) reached the consensus threshold among all participants. Ten items (18%) did not get the consensus threshold.

### 3.2 Consensus levels by the key topics: comorbidities, early relapse, and *ab initio* metastatic tumor

#### 3.2.1 Comorbidities: First-line therapeutic choices in patients with HR+/HER2- MBC and multiple comorbidities

The level of agreement/disagreement and consensus outcomes on items regarding the role of comorbidities in therapeutic choices for HR+/HER2- MBC patients receiving first-line therapy for their disease are shown in **Table 1**.

For most items, 12/16 (75%) reached a consensus. Three items (19%) did not get a consensus, while one (6%) reached an agreement among the local oncologists but not the faculty.

The main area of debate and disagreement among the faculty and the local oncologists was on the differential use of CDK4/6i according to patients’ age, the best ET, i.e., fulvestrant vs aromatase inhibitors (AIs), to be combined with CDK4/6i, and the optimal timing for *PIK3CA* mutation evaluation on tissue or liquid biopsy.

Moreover, rates of agreement not reaching the consensus threshold within the faculty and the local oncologists were 57% vs 60%; 60% vs 64% for item 5 (In the presence of a very old patient (75 years old), is there a preference for a CDK4/6i over the others?) and item 9 (In patients with multiple comorbidities, in the absence of a *PIK3CA* mutation, is the first-line treatment with ribociclib in association with fulvestrant to be preferred over AI + CDK4/6i?), respectively.

With regards to statement 7, which addressed the timing of *PIK3CA* evaluation through biopsy, on item 7.1 (At the first diagnosis of metastatic disease, to define the therapeutic course of the first and second line), rates of the agreement were 64% within the faculty and 92% among the local oncologists, thus reaching the consensus threshold in the latter but not in the former.Finally, on item 7.3 (before starting the second-line therapy, even if previously carried out and negative), the agreement rates were 64% within the faculty, while the disagreement rates were 64% among the local oncologists, thus not reaching the consensus threshold.

For all the other items included in the survey, levels of agreement or disagreements among faculty and local oncologists were quite high, ranging from 80 to 100%, as shown in **Table 1**.

#### 3.2.2 Early relapse: First-line therapeutic choices in early recurring HR+/HER2- MBC patients

The level of agreement/disagreement and consensus outcomes on items regarding comorbidities are shown in **Table 2**. Out of the 21 items, 19 (90%) reached an overall consensus. Only one item (5%) did not reach consensus in both groups, and one (5%) did not reach consensus only among the local oncologists. Furthermore, in statement 2 (In relation to the previous question, in clinical situations where it was decided to use fulvestrant + CDK4/6i, the choice could fall on), the option “abemaciclib” and “ribociclib” reached a level of agreement of 100% vs 92% within the faculty and the local oncologists respectively. For the option “palbociclib,” the level of agreement was 71% and 64% by the faculty and local oncologists, respectively, thus reaching the consensus threshold in the former but not in the latter.

In the statement 8 (In relation to the previous question, in clinical situations where the decision was made to use a luteinizing hormone-releasing hormone antagonist (aLHRH) + fulvestrant + CDK4/6i, the choice could fall on), there was a level of agreement of 92% vs 80% for “abemaciclib”, 64% vs 48% for “palbociclib,” which did not reach a consensus threshold, and 100% vs 88% for “ribociclib” by the faculty and the local oncologists respectively.

For all the other items included in the survey, levels of agreement or disagreements among faculty and local oncologists were quite high, ranging from 90 to 100%, as shown in **Table 2** for most of the items.

#### 3.2.3*Ab initio* metastatic tumor: first-line therapeutic choices in *ab initio* HR+/HER2- MBC patients

Statements related to the management of *ab initio* MBC were the most controversial. The level of agreement and consensus are shown in **Table 3**. Out of 19 items, 14 (74%) reached consensus, and five (26%) did not reach overall consensus.

The main area of debate and disagreement among the faculty and local oncologists concerned the choice of therapy (i.e., AI + any CDK4/6i or fulvestrant + ribociclib) that should be used in *ab initio* metastatic patients without *PIK3CA* mutations (item 4) and on the best therapeutic approach, ET + CDK4/6i be proposed over a “rescue” chemotherapy in case of visceral crisis (item 5). Moreover, within the statement 4 (In *ab initio* metastatic patients without *PIK3CA* mutations, the first-line treatment with fulvestrant + ribociclib combination is to be preferred over the combination of AI + any CDK4/6i in case of), none of the options except 4.4 reached at least a partial consensus. For the options “exclusively bone disease,” “oligometastatic disease,” and “visceral disease, regardless of disease burden,” levels of agreement were 64% vs 64%, 50% vs 56%, and 64% vs 64% by the faculty and local oncologists, respectively, thus not reaching the consensus threshold at all. “Visceral disease, with high disease burden” reached a level of agreement of 64% by the faculty and 72% among local oncologists, thus achieving the consensus threshold in the latter but not in the former.

Statement 5 (In case of visceral crisis and *ab initio* metastatic disease with high hormonal receptor expression, can an ET + CDK4/6i be proposed over a “rescue” chemotherapy)? reached an agreement level of 87% within the faculty. There was a disagreement level of 64% among local oncologists, thus reaching the consensus threshold in the former but not in the latter.

For all the other items included in the survey, levels of agreement or disagreements among faculty and local oncologists were quite high, ranging from 90 to 100%, as shown in **Table 3** for most of the items.

## 4 Discussion

Despite recent findings and breakthrough therapies in treating HR+/HER2- MBC, some topics remain controversial mainly for the lack of level I evidence to drive clinical decisions. For instance, patients with comorbidities or visceral crises are poorly represented in pivotal clinical studies, given the impossibility of conducting proper interventional therapies. In this scenario, evidence generated by a consensus of experts may be useful. By a modified Delphi approach, the present study identifies the most debated topics in HR+/HER2- MBC setting, quantifies the level of discordance or agreement among oncologists dedicated to breast cancer and oncologists not dedicated to breast cancer specifically, and finally tries to draw reasonable guidelines mainly based on expert opinion and everyday practice.

The strength of this study, by combining the NGT with the Delphi Survey, allows clinicians to share their own opinions based on personal experience and to work towards an integration of such opinions. Overall, our work showed a high consensus on most of the proposed topics. Statements that reached the consensus threshold may be reliable suggestions for routine clinical practice.

### 4.1 First-line therapeutic choices in patients with comorbidities

In patients with multiple comorbidities, the first line of choice in bone and visceral disease, regardless of disease burden, is the association of ET with a CDK4/6i or either abemaciclib, palbociclib, or ribociclib (**Table 1**, items 1.1–2.3, 5). Recent real-world experiences have shown that dose reduction, especially in patients older than 70 years and those with comorbidity, did not affect the progression-free survival (PFS) and overall survival (OS) (23). The board did not opt for a routine CDK4/6i dose reduction in this group of patients, even if they were over the age of 75 years (**Table 1**, items 3, 4, 6). Toxicity profile and pharmacological interactions with other drugs must be carefully monitored in this group of more fragile patients.

One of the most controversial topics was the appropriate timing for evaluating the *PIK3CA* mutation. Recent data show that in the presence of a *PIK3CA* mutation, alpelisib + fulvestrant can be considered standard treatment after progression by AIs, as demonstrated in the SOLAR-1 study (24). However, to date, there is no reimbursement for patients in Italy for alpelisib + fulvestrant after progression by a CDK4/6i + AI, even though the oncological community is very confident about the activity of alpelisib in such patient populations due to the recent findings of the BYLieve phase II study (25). In the BYLieve study, among patients following progression with or after previous therapy, including CDK4/6i, 50% of patients were alive without disease progression at 6 months. It is well-agreed that the *PIK3CA* test should be assessed before starting second-line therapy, if not previously performed, and that liquid biopsy is a valid alternative to tissue biopsy when the latter is not feasible (**Table 1**, items 7.2, 8). However, local oncologists appear to be more prone to carry out the biopsy at the first diagnosis to guide first- and second-line treatments compared with the faculty (**Table 1**, item 7.1). Interestingly, repeating the biopsy before starting the second-line therapy to revaluate *PIK3CA* after a previous negative result is even more debated. In statement 7.3, the consensus is not reached and, importantly, agreement levels on the same statement go in the opposite direction: 64% of faculty agreed, and 64% of local oncologists disagreed with the repetition of a second biopsy for *PIK3CA* determination.

The leading causes for the observed discordance may be the following: among local oncologists, difficulties in performing tissue biopsy at the metastatic site or repeating liquid biopsy, which is not a standard of care at the moment, and among faculty, the low probability of seeing modulation of phosphatidylinositol 3-kinase (*PI3KCA*) mutation during the progressions, as documented in other breast cancer subtypes (26), and the low performances of patients in more advanced therapeutic lines.

Our survey also shows uncertainty in prescribing ribociclib + fulvestrant as first-line treatment in the absence of the mutation (**Table 1**, statements 9–10). The reluctance in fulvestrant use in place of AIs is explained by recent MONALEESA 2 data showing an important survival advantage for patients on ribociclib + letrozole vs letrozole alone (hazard ratio [HR] = 0.76, in favor of ribociclib + letrozole; 95% CI: 0.63–0.93; p=0.004; median OS 63.9 vs 51.4 months in ribociclib + letrozole vs letrozole alone, respectively). Importantly, the OS continued to increase with longer follow-ups. At 4 years, the absolute improvement was 5.7% favoring ribociclib; this rate increased to 8.4% at 5 years and 12.2% at 6 years. At 6 years, 44.2% of the patients in the ribociclib arm were alive, compared with 32% of those given ET alone. Consistently with MONALEESA 2, data from MONALEESA-7 (14), also showed a PFS and OS advantage in premenopausal women treated with ribociclib + ET (tamoxifen or AI+ aLHRH) vs ET alone, further strengthen confidence in the use of AIs in association to CDK4/6i regardless the menopausal status in the first-line setting.

### 4.2 First-line therapeutic choices in early recurring HR+/HER2- MBC

First-line therapeutic choices in early recurring HR+/HER2- MBC were the most agreed-on topic. Notably, there was a complete agreement on not considering chemotherapy an option as first-line treatment in early recurring HR+/HER2- MBC patients, regardless of menopausal status, age, and metastatic sites (bone or visceral) disease (**Table 2**, items 3, 9 and 11).

Even for those who had received tamoxifen monotherapy in premenopausal patients, aLHRH + fulvestrant + CDK4/6i was the preferred first-line treatment. However, the first choices CDK4/6i in this setting were abemaciclib and ribociclib, with the latter to be preferred in case of a very early relapse at the beginning of adjuvant ET (**Table 2**, items 1–2.3 and 4–6). No consensus was met on palbociclib. The MONALEESA-7 study (14), including about 30% of the young patients with progressive disease in less than 12 months from previous neo/adjuvant ET, may be considered the main reason for the survey outcome.

In postmenopausal patients, the combination of fulvestrant and CDK4/6i, such as abemaciclib or ribociclib, is the first-line treatment of choice, regardless of age. In elderly patients (75 years-old), though there are no specific studies in this population, monotherapy with fulvestrant was strongly discouraged. The phase II study, FACILE, which is enrolling 70-year-old patients on ribociclib + letrozole as the first line, is currently ongoing and will provide efficacy and safety data in the future (27) As for the other settings, doubts remain on the efficacy of palbociclib compared to the other two CDK4/6i (**Table 2**, items 7-8, 10.1–10.3). Most of the participating oncologists in both groups did not consider palbociclib the first choice in this setting. ribociclib and abemaciclib, which showed improvement of OS in endocrine-resistant patients (18, 20), were considered more appropriate. Disagreement on the use of palbociclib may be due to results of the PEARL Study that did not show superiority in terms of PFS of palbociclib + fulvestrant vs capecitabine in first-line endocrine-resistant patients (28) or to the disappointing results of palbociclib in the early disease (29, 30).

### 4.3 First-line therapeutic choices in *ab initio* metastatic HR+/HER2- MBC

In patients with *ab initio* HR+/HER2- MBC, the association of CDK4/6i + ET was demonstrated to be superior to ET alone (12, 15, 17, 19, 20). There is no direct evidence comparing first-line ET + CDK4/6i in the AI-sensitive population versus chemotherapy. However, indirect evidence from a recent network meta-analysis clearly shows the superiority of CDK4/6i + AI over chemotherapy in the first-line treatment of HR+/HER2- MBC (31).

Before the CDK4/6i era, several international guidelines have suggested ET as the preferred first-line therapy in MBC patients with an endocrine-sensitive disease (7–11). However, several studies showed chemotherapy was still very popular in this setting (32, 33). Data from the present survey shows a high concordance between the faculty and local oncologists in considering ET + CDK4/6i the preferred first-line treatment over chemotherapy or ET alone in all the patient subgroups (bone only, oligometastatic, visceral disease, and high-burden visceral disease) and, importantly, in patients older than 75 years too. However, they are expected to have a higher prevalence of comorbidity and are less represented in clinical trials (**Table 3**, items 1–3.4). Agreement on the use of ET + CDK4/6i in the elderly patient population is very relevant as these patients could be undertreated with ET alone because of age. It is important to notice that no relevant incremental toxicity or reduced clinical benefit has been shown in older patients, though data are sparse and not exhaustive (34). A high level of agreement was also reached on the timing of *PIK3CA* mutation evaluation, on tissue or liquid biopsy analysis, best before starting first-line treatment, to better plan first- and second-line treatment strategy (**Table 3**, items 6 and 7). In *PIK3CA* mutation carriers, both faculty and local oncologists agreed on the use of AIs + CDK4/6i in the first line and fulvestrant + the PIK3CA inhibitor alpelisib (**Table 3**, item 7) in the second line according to SOLAR-1 study (23) and BYLieve study (25). However, currently, the European and Italian regulatory agencies (i.e., EMA and AIFA) limited the use of alpelisib to *PIK3CA*-mutated MBC patients progressing on a previous endocrine monotherapy, excluding de facto patients treated with first-line CDK4/6i (35, 36). At the same time, FDA approval for the drug is generically “following progression on or after an endocrine-based regimen” (37).

No consensus in our study was met regarding the best endocrine therapy to use upfront in the first-line setting in patients without the *PIK3CA* mutation (**Table 3**, items 4.1–4.4). In the MONALEESA-3 trial (20), the combination of fulvestrant + ribociclib showed a statistically significant increase of PFS and OS over fulvestrant + placebo both in first-line (endocrine sensitive) and second-line (endocrine-resistant) HR+/HER2- MBC. Furthermore, the updated descriptive PFS analysis of patients treated in the first line demonstrated a longer PFS in the ribociclib arm compared to the previously reported PFS for first-line treatment with CDK4/6i + AI in postmenopausal patients (38–40). The authors claimed that PFS and OS data might support the consideration of ribociclib plus fulvestrant as initial therapy in patients with advanced disease (38). However, cross-trial comparisons should be made with caution. Moreover, the only randomized study directly comparing first-line fulvestrant + CDK4/6i (palbociclib) with AI + CDK4/6i (palbociclib) showed no statistical difference in terms of PFS, objective response rate, and 3-year OS rate (41). Therefore, our study’s unmet agreement on this topic could be due to current uncertainty on the best first-line treatment strategy and the idea that fulvestrant may be a good treatment option in more endocrine-resistant diseases as in second and further lines (42).

Finally, local oncologists could not reach a consensus on using CDK4/6i + ET instead of rescue chemotherapy in patients in visceral crisis (**Table 3**, item 5). However, a positive consensus was reached among the faculty. International guidelines suggest chemotherapy over ET for patients with HR+/HER2- MBC in case of visceral crisis (11, 43, 44) as a faster antitumor activity is needed. However, data from registration trials clearly show that CDK4/6i have rapid antitumor activity and elicit objective responses comparable (or higher) to chemotherapy (12–20). However, patients with visceral crisis were excluded from these trials; therefore, direct evidence of CDK4/6i activity in these patients is lacking. The 5th ESO-ESMO international consensus guidelines for advances breast cancer (ABC 5) (11) reviewed the definition of visceral crisis, estimating its occurrence in about 10–15% of first-line MBC cases, and defined that this clinical scenario requires “the use of the most rapidly efficacious therapy, which is not necessarily chemotherapy in all situations,” paving the way, *de facto*, to CDK4/6i treatment. In our study, the faculty reached a positive consensus on the possibility of proposing CDK4/6i in case of visceral crisis, probably due to a higher clinical experience and confidence in this drug class, believing the rapid response to be similar to chemotherapy, but with lower and more manageable side effects. On the contrary, local oncologists did not find consensus (either in agreement or disagreement) on this topic, demonstrating the presence of an intra-group debate about the best treatment choice in this challenging clinical scenario.

### 4.4 Limitations

This work has some limitations. As a consensus work, it cannot produce novel empiric data. During the Delphi survey, voters could not give a position of “no opinion” or comment on the pertinence of the statements drafted by the SB. Finally, being limited to the Italian setting, results may not be generalized to other countries since the clinical practice can vary due to different resources and regulations.

## 5 Conclusion

Despite the interesting recent advances in the treatment of HR+/HER2- MBC, to date, many grey areas remain on the topic, both due to lack of data and because data are derived from *post hoc* analyses of randomized clinical trials. From this perspective, our study aimed to measure the levels of agreement and disagreement and consensus status on such grey areas. Using a structured methodology, such as the NGT and the Delphi Survey, allowed participants to share their own opinions rising from their personal experience and work towards integrating such opinions. Overall, results from the Delphi Survey show an almost evident agreement between oncologists working in referral vs local spoke oncological hospitals. Where consensus was not met, possible explanations in light of differences in clinical practice and recent literature findings are provided in the Discussion. Items in **Tables 1**–**3** and relative elucidation can be of use in guiding treatment in case of therapeutic decisions in HR+/HER2- MBC patients with multiple comorbidities, early recurring disease, and *ab initio* metastatic disease.

## Data availability statement

The original contributions presented in the study are included in the article/supplementary material. Further inquiries can be directed to the corresponding author.

## Ethics statement

Ethical review and approval was not required for the study on human participants in accordance with the local legislation and institutional requirements. Written informed consent for participation was not required for this study in accordance with the national legislation and the institutional requirements.

## Author contributions

Study conception and design: AF, EB, GA, and GBu. Collection, interpretation, and discussion of data from the Delphi survey and literature: all authors. Manuscript drafting: AF, EB, GA, and GBu. manuscript editing: all authors. All Authors gave their approval to submit.

## Funding

The work was funded with a non-conditioning grant by Novartis. The funder was not involved in the study design, collection, analysis, interpretation of data, the writing of this article or the decision to submit it for publication.

## Acknowledgments

We would like to thank Over SRL for the organization and coordination of the project, and Fabio Perversi, Aashni Shah and Valentina Attanasio (Polistudium SRL, Milan, Italy), for medical writing and editorial assistance. We also wish to acknowledge all the member of the Delphi Panel who participated in the survey: Arrivas Bajardi Eugenia, Bighin Claudia, Botticelli Andrea, Catania Giovanna, Fabbri Agnese, Fontana Andrea, Garrone Ornella, Leonardi Vita, Meattini Icro, Mura Silvia, Pantano Francesco, Ricciardi Giuseppina Rosaria Rita, Rizzo Gian Piero, Sanò Maria Vita, Sartori Donata, Sidoni Tina, Coltelli Luigi, Cortesi Laura, Ferro Antonella, Losurdo Agnese, Minisini Alessandro, Musolino Antonino, Palazzo Antonella, Pedani Fulvia, Riva Francesca, Schirone Alessio.

## Conflict of interest

AF received honoraria or speakers’ fee from Roche, MSD, AstraZeneca, Pfizer, Novartis, Eli-Lilly, Dompè, Eisai and GSK. EB received honoraria or speakers’ fee from MSD, AstraZeneca, Celgene, Pfizer, Helsinn, Eli-Lilly, BMS, Novartis and Roche. EB is supported by the Associazione Italiana Ricerca Cancro (AIRC grants n. IG 20583). GBu received honoraria or speakers’ fee from Novartis, GSK, Eli-Lilly, Pfizer, AstraZeneca, Roche and Genetic. GBi received Consultancy/Honorarium fees from Roche, Pfizer, AstraZeneca, Lilly, Novartis, Neopharm Israel, Amgen, MSD, Chugai, Sanofi, Daiichi Sankyo, EISAI, Gilead, Seagen and Exact Science. GC was on the advisory board for Pfizer, Novartis, Lilly, Seagen, Amgen, Roche, AstraZeneca, Daichii Sankyo, Celcuity, Veracyte and Gilead (outside the submitted work). MDL received honoraria or speakers’ fee from AstraZeneca, Amgen, Celgene, Daiichi Sankyo, Eisai, Eli Lilly, Exact Science, Gilead, MSD, Novartis, Pfizer, Pierre Fabre, Roche and Seagen. SDP has received personal fees (participation on advisory board and/or speakers bureau) from Novartis, Roche, Celgene, Bristol Myers Squibb, AstraZeneca, Pfizer, Lilly, Eisai, Seagen, Daiichi Sankyo, Clovis, GSK, MSD, Gilead and Exact Sciences. LDM has received personal fees from Novartis, Pfizer, Roche, Eli Lilly, Astra Zeneca,Pierre Fabre, Eisai, Daiichi Sankyo, Seagen, Gilead, Exact Sciences and Ipsen. VG received honoraria or speakers’ fee from Eli Lilly, Novartis, MSD, GSK, Gilead, EISAI and Amgen. DG received honoraria or speakers’ fee from Eli Lilly, Novartis, Pfizer, AstraZeneca, Roche and Eisai. LL received honoraria or speakers’ fee from Novartis, Eli Lilly, Pfizer, Roche, AstraZeneca and MSD. VL received honoraria or speakers’ fee from Amgen, AstraZeneca, Daichii Sankyo, Celgene, Eisai, Eli Lilly, Gilead, GSK, Ipsen, MSD, Novartis, Pierre-Fabre, Pfizer, Roche, Seagen and Takeda. FP received grants/research support from AstraZeneca, Eisai and Roche and the receipt of honoraria or consultation fees from Amgen, Astrazeneca, Daichii Sankyo, Celgene, Eisai, Eli Lilly, Gilead, GSK, Ipsen, MSD, Novartis, Pierre-Fabre, Pfizer, Roche, Seagen, Takeda and Viatris; all disclosures are outside the submitted work. PV received honoraria from AstraZeneca; Eli Lilly; Gilead; GSK; Istituto Gentili; Novartis; Pfizer; Roche and Teva. PV received research funding from Novartis and Pfizer. AZ reports personal fees and non-financial support from Novartis, AstraZeneca, Lilly, Pfizer, Daiichi Sankyo, MSD, Roche, Seagen, Exact Sciences, Gilaed and Istituto Gentili; all disclosures are outside the submitted work. GA received consulting fees from Roche, Pfizer, Lilly, MSD, AstraZeneca and Novartis.

The remaining authors declare that the research was conducted in the absence of any commercial or financial relationships that could be construed as a potential conflict of interest.

## Publisher’s note

All claims expressed in this article are solely those of the authors and do not necessarily represent those of their affiliated organizations, or those of the publisher, the editors and the reviewers. Any product that may be evaluated in this article, or claim that may be made by its manufacturer, is not guaranteed or endorsed by the publisher.
